# Robust combination of liver stereotactic body radiotherapy modulates pharmacokinetics of sorafenib toward preferable parameters

**DOI:** 10.1038/s41598-020-66583-9

**Published:** 2020-06-12

**Authors:** Chen-Hsi Hsieh, Yu-Jen Chen, Tung-Hu Tsai, Li-Ying Wang, Hung-Chi Tai, Hsiang-Ling Huang, Yu-Chuen Huang

**Affiliations:** 1 Institute of Traditional Medicine, School of Medicine, National Yang-Ming University, Taipei, Taiwan; 2 Faculty of Medicine, School of Medicine, National Yang-Ming University, Taipei, Taiwan; 3 Division of Radiation Oncology, Department of Radiology, Far Eastern Memorial Hospital, Taipei, Taiwan; 4 Department of Radiation Oncology, Mackay Memorial Hospital, Taipei, Taiwan; 5 Department of Medical Research, Mackay Memorial Hospital, Taipei, Taiwan; 6 Department of Chemical Engineering, National United University, Miaoli, Taiwan; 7 School and Graduate Institute of Physical Therapy, College of Medicine, National Taiwan University, Taipei, Taiwan; 8 Physical Therapy Center, National Taiwan University Hospital, Taipei, Taiwan; 9 Department of Medical Research, China Medical University Hospital, Taichung, Taiwan; 10 School of Chinese Medicine, China Medical University, Taichung, Taiwan

**Keywords:** Hepatocellular carcinoma, Drug regulation

## Abstract

To evaluate the effect and mechanism of radiotherapy (RT)–sorafenib pharmacokinetics (PK) in different regimens with conventional or high dose irradiation. Between February 2012 and December 2018, 43 patients with portal vein tumor thrombosis treated with sorafenib plus conventional RT (58%) or stereotactic body radiation therapy (SBRT, 42%) were retrospectively reviewed. *In vivo* and *in vitro* studies of concurrent and sequential RT with sorafenib were designed. SBRT resulted in a 3-fold increase in complete recanalization compared to conventional RT group (28% vs. 8%, *p* = 0.014). Compared to the control group, the area under the concentration vs. time curve (AUC) of sorafenib was increased in the concurrent RT_2Gy_ and RT_9Gy_ groups and the sequential RT_9Gy_ group by 132% (*p* = 0.046), 163% (*p* = 0.038) and 102% (*p* = 0.018), respectively; and was decreased by 59% in the sequential RT_2Gy_ group (*p* = 0.036). Sequential RT_2Gy_ and RT_9Gy_ increased CYP3A4 activity by 82% (*p* = 0.028) and 203% (*p* = 0.0004), respectively, compared to that with the corresponding concurrent regimen. SBRT produced better recanalization than conventional RT with sorafenib. The AUC of sorafenib was modulated by RT. P-gp expression was not influenced by RT. The sequential RT regimen increased CYP3A4 activity that may increase the RT-sorafenib synergy effect and overall sorafenib activity. The biodistribution of sorafenib was modulated by local RT with the different regimens.

## Introduction

Hepatocellular carcinoma (HCC) is one of the most prevalent solid tumors worldwide^1^. Less than 30% of patients are eligible for curative treatments^2^, and most are incurable^3,4^. Stereotactic body radiation therapy (SBRT) is an alternative treatment to ablation/embolization techniques or can be used when these techniques either fail or are contraindicated for HCC^5,6^. Additionally, SBRT exhibits a dose-response relationship for local control and overall survival in HCC patients^7,8^. Sorafenib (Nexavar, Bayer Pharma AG, Berlin, Germany) is an oral multikinase inhibitor that targets the Raf/mitogen-activated protein kinase (MAPK)/extracellular-signal-regulated kinase (ERK) signaling pathway to induce tumor cell apoptosis in HCC^9,10^.

The combination of oral sorafenib with RT or SBRT may exhibit synergy for inhibiting tumor growth^11^. However, toxicity of the SBRT-sorafenib combination for HCC with a high effective volume of irradiated liver has been noted^12,13^. Recently, RT has been shown to modulate the systemic pharmacokinetics (PK) of anticancer drugs and affect the composition of the microenvironment^14–17^. These lines of evidence suggest that interactions between sorafenib and RT may modulate the PK of sorafenib. However, the time schedule and dose of RT for use in combination with sorafenib are controversial.

The present study was designed to evaluate the possible mechanism of the RT- PK of sorafenib with different time schedules and doses in both *in vitro and in vivo* studies and assess the clinical response to provide suggestions for clinical applications.

## Methods

### Materials and reagents

Dimethyl sulfoxide (DMSO), 3-(4′,5′-dimethylthiazol-2′-yl)-2,5-diphenyltetrazolium bromide (MTT), cyclosporin A (CsA), ketoconazole, digoxin, ethyl paraben, acetonitrile (liquid chromatography [LC] grade), methanol and ethyl acetate (LC grade) were purchased from Merck (Merck Ltd., Taiwan). Sorafenib was purchased from Santa Cruz Biotechnology, Inc. (Dallas, TX, USA). Dulbecco’s modified Eagle’s medium (DMEM), fetal bovine serum, 100 IU/mL penicillin, 100 mg/mL streptomycin, and 1% nonessential amino acids were purchased from Biological Industries (Cromwell, CT, USA). Milli-Q plus water (Millipore, Bedford, MA, USA) was used for all preparations.

### HCC patients with portal vein tumor thrombosis (PVTT) treated by sorafenib with conventional RT or SBRT

#### Patient selection

We retrospectively reviewed HCC patients with PVTT who received sorafenib and RT at the Far Eastern Memorial Hospital between February 2012 and December 2018. The need for informed consent was waived by the Institutional Review Board of the Far Eastern Memorial Hospital (FEMH-IRB-108025-E) and retrospective data were collected after receiving approval from the Institutional Review Board of the Far Eastern Memorial Hospital (FEMH-IRB-108025-E). All research was performed in accordance with relevant guidelines and regulations. All tumors were staged according to the American Joint Committee on Cancer (AJCC) Cancer Staging Manual, 7^th^ edition. A total of 90 HCC patients with PVTT were identified. Patients who were not treated with sorafenib (n = 32), for whom the treatment target did not include PVTT (n = 2), or who did not undergo subsequent abdominal computed tomography (CT) after RT treatment (n = 13) were excluded; the remaining 43 patients were enrolled. The patients who were treated with a radiation fraction size of <5 Gy and those treated with ≥5 Gy were grouped as the conventional and the SBRT group, respectively.

### *In vitro* studies

#### Cell viability assay

Huh-7 cells were plated in 96-well plates (1 × 10^4^ cells/well) in 100 μL of serum-containing medium and allowed to grow for 1 day. Sorafenib concentrations of 0, 2.5, 5, 10 and 20 μmol/L (μM) were added to the plates 1 hour (hr) after irradiation (concurrent group) or 24 hr after irradiation (sequential group) with sham RT (RT_0Gy_), 2 Gy (RT_2Gy_) or 9 Gy (RT_9Gy_). After 1 day, 15 μL of 5 mg/mL MTT was added and incubated for 4 hr. The absorbance values were read with a microplate reader at a wavelength of 570 nm and a reference wavelength of 630 nm.

#### Effects of RT on P-glycoprotein (P-gp) activity

A rhodamine 123 (Rho-123, Thermo Fisher Scientific, Pittsburgh, PA, USA) transport assay was performed to observe the effects of RT and sorafenib on the activity of P-gp as described previously^18,19^. In brief, Huh-7 cells were seeded in a 6-well plate. RT_0Gy_, RT_2Gy_, or RT_9Gy_ was applied. At 1 hr or 24 hr after RT, ketoconazole (a P-gp inhibitor), digoxin (a P-gp substrate) and DMSO were added to the corresponding wells and incubated at 37 °C. The existing medium was replaced with 20 μM Rho-123 solution and incubated for 1 hr. Then, the cells were analyzed (10000 cells/sample) to measure Rho-123 accumulation with a FACSCalibur flow cytometer (excitation (Ex) = 515 nm, emission (Em) = 545 nm; Becton Dickinson, San Jose, CA, USA). The data were analyzed with CellQuest software (Becton Dickinson, San Jose, CA, USA).

#### Effects of RT on P-gp expression—Western blotting

The effect of RT on P-gp protein expression was initially assessed in cell lysates. Cells were harvested and washed twice with cold PBS and were then resuspended and lysed in cell lysis buffer at 4 °C for 30 min. Lysates were centrifuged for 10 minutes (min) at 12000 rpm, and supernatants were stored at −80 °C as whole-cell extracts. Total protein concentrations were determined by a Bradford assay. Proteins were separated on 10% SDS-PAGE gels and transferred to polyvinylidene difluoride membranes. Membranes were blocked with 5% BSA and incubated with the indicated primary antibodies. Corresponding horseradish peroxidase-conjugated secondary antibodies against each primary antibody were used. Proteins were detected using chemiluminescence detection reagents.

#### Effects of RT and NF-κB inhibition on P-gp activity

The peptide SN50 inhibits nuclear translocation of NF-κB. SN50M was used as a negative control^20^. In brief, Huh-7 cells were pretreated with 20 μM SN50 or SN50M (Enzo Life Sciences, Inc., Farmingdale, NY, USA) for 1 hr and were then irradiated. At 1 hr or 24 hr after RT, the existing medium was replaced with 20 μM Rho-123 and incubated for 1 hr. The cells were analyzed (10000 cells/sample) to measure Rho-123 accumulation with a FACSCalibur flow cytometer (Ex = 515 nm, Em = 545 nm; Becton Dickinson, San Jose, CA, USA). The data were analyzed with CellQuest software (Becton Dickinson, San Jose, CA, USA).

### *In vivo* study

#### Animals and sample preparation

Adult male Sprague-Dawley rats (body weight, 300 ± 20 g) were provided by the Laboratory Animal Center at National Yang-Ming University (Taipei, Taiwan). Rats were housed in a specific pathogen-free environment with free access to food (Laboratory Rodent Diet 5001, PMI Nutrition International, LLC, MO, USA) and water. All experimental animal surgical procedures were reviewed and approved by the animal ethics committee of Far Eastern Memorial Hospital, New Taipei city, Taiwan (FEMH-103-01-27-A). All experiments were performed in accordance with relevant guidelines and regulations.

#### Irradiation technique

**Figure 1:**
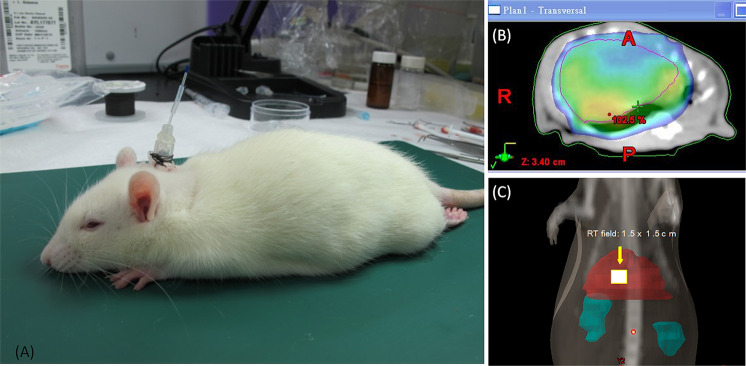
(**A**) Freely moving rat model. (**B**) Whole-liver irradiation. (**C**) SBRT 1.5 × 1.5 cm in the central area of the liver. The irradiated field of the liver in Sprague-Dawley rats was targeted by computed tomography.

A freely moving rat model was designed for the current study^21^ (Fig. 1A). Experimental rats were anesthetized with 1 g/mL urethane and 0.1 g/mL α-chloralose (1 mL/kg by intraperitoneal [i.p.] injection), and polyethylene tubes were then implanted in the right jugular vein for intravenous (i.v.) fluid administration. The right jugular vein was catheterized for blood sampling. The catheter traversed the subcutaneous tissue and was fixed in the dorsal neck region. After surgery, the rats were placed in an experimental cage and allowed to recover for 1 day. During the recovery period, the rats were kept warm under a light. Then, the rats were anesthetized and immobilized on a board while undergoing CT for localization of the whole liver (conventional technique, Fig. 1B) or a central area of 1.5 × 1.5 cm (SBRT technique, Fig. 1C).

#### Drug delivery with RT under different time schedules and doses

The suggested dosage of sorafenib to be administered concurrently with SBRT is 400 mg daily^12^. We established a dosage of sorafenib for the rats as 40 mg/kg/day according to the following formula: human equivalent dose (HED, mg/kg) = animal dose (mg/kg) × animal km/human km^22^. The rats were randomly divided into five groups of six rats per group. The study group included (A) a sham group, treated with sorafenib and RT_0Gy_; two concurrent groups, treated with sorafenib 1 hr after (B) RT_2Gy_ or (D) RT_9Gy_; and two sequential groups, treated with sorafenib 24 hr after (C) RT_2Gy_ or (E) RT_9Gy_.

#### Pretreatment with CsA for drug delivery with RT under different time schedules and doses

The effects of CsA^23^, an inhibitor of CYP3A and P-gp, were investigated in rats treated with sorafenib. The rats were pretreated with CsA (20 mg/kg, i.p.). After 30 min, sorafenib (40 mg/kg) was administered intragastrically. Blood samples were obtained according to a preset schedule.

#### Sample preparation

Blood samples (150 µL) were withdrawn from the jugular vein with a fraction collector at 15, 30, 45, and 60 min and at 1.5, 2, 2.5, 3, 3.5 and 4 hr after drug administration. The blood samples were immediately centrifuged at 4200 × g for 10 min. The resulting plasma (50 µL) was added to 1 mL of ethyl acetate in a clean tube, vortexed for 5 min, and centrifuged at 5900 × g for 10 min. After centrifugation, the upper organic layer containing ethyl acetate was transferred to a new tube and evaporated to dryness under a nitrogen flow.

#### High-performance liquid chromatography (HPLC)

Chromatographic analysis was performed on a Model LC-20DAD HPLC system (Shimadzu, Tokyo, Japan) equipped with a Model SPD-M20A wavelength UV detector, a SIL-20AC autosampler, and an LC Solution data processing system. A Waters Acquity C^18^ column (50 × 2.1 mm, particle size 1.7 μm, Eclipse XDB; Agilent, Palo Alto, CA, USA) was used for HPLC separation. The mobile phase consisted of potassium dihydrogen phosphate (10 mM, pH = 3) and acetonitrile (55:45, v/v). The flow rate was set at 0.2 mL/min, and the injection volume was 5 μL. Sorafenib and diethylstilbestrol (the internal standard [IS]) were detected at 265 nm. A peak controlled spectrum recording was selected with a range of 190–300 nm.

#### Plasma and biliary excretion after RT or SBRT followed by i.v. administration of Rho-123

For i.v. administration, Rho-123 was used at a final concentration of 0.2 mg/mL. For the collection of blood and bile at designated intervals over 120 min, rats were anesthetized as described above. Rho-123 (0.2 mg/kg) was administered intravenously at 10 min or at 24 hr after irradiation.

An equal volume of methanol was added to 100-μL aliquots of bile and plasma sample specimens for deproteinization. The mixture was vortexed for 15 s and centrifuged at 12000 × g for 10 min. Then, 100-μL aliquots of the supernatant were transferred to the wells of a 96-well microplate, and the Rho-123 concentration in the samples was measured by fluorometric detection (Ex = 485 nm, Em = 527 nm).

#### Determination of cytochrome P450 3A4 (CYP3A4) activity in hepatic microsomes after RT or SBRT

Liver tissues from the different groups of rats were removed, homogenized and centrifuged at 10000 × g for 15 min at 4 °C. The activity of microsomal CYP3A4 was measured using a CYP3A4 Activity Assay Kit (Biovision, Inc., Milpitas, CA, USA). For liver postmitochondrial fractions, the initial protein concentration was 1–2 mg/mL. In addition to the test sample wells, background control (no enzyme) and inhibitor control (30 µM ketoconazole) wells were prepared. The volumes of test sample, inhibitor control and positive control wells were adjusted to 70 µL/well. The plate was incubated for 5–10 min at 37 °C to allow ketoconazole to interact with CYP3A4 in the absence of P450 catalytic turnover. Within 1 min, the fluorescence at Ex/Em = 535/587 nm was measured in kinetic mode for 30–45 min at 37 °C.

#### Organ distribution

Six hours after sorafenib administration (40 mg/kg, po), blood samples were collected as described above. The brain, liver, heart, spleen, lung and kidney were collected and weighed. These collected samples were stored at −20 °C until analysis.

#### Organ samples

Thawed organ samples were homogenized in 50% aqueous acetonitrile (sample weight:volume ratio, 1:5), and the homogenate was then centrifuged at 13000 × g for 10 min at 4 °C. The supernatant was collected, placed in brown Eppendorf tubes, and stored at −20 °C until analysis. In brief, each organ sample (50 μL) was combined with 150 μL of IS solution (diethylstilbestrol) for protein precipitation. Finally, 20 μL of filtrate was injected into the HPLC system for analysis.

### PK and data analysis

Pharmacokinetic parameters, including the area under the concentration vs. time curve (AUC), terminal elimination phase t_1/2_, Cmax, MRT, total plasma clearance and Vss, were calculated using the PK calculation software WinNonlin Standard Edition, version 1.1 (Scientific Consulting, Apex, NC, USA) using a compartmental method.

### Calculations and data analysis

All statistical calculations were performed with Statistical Package for the Social Sciences (SPSS) for Windows, version 20.0 (SPSS, IBM, USA). The results of *t* tests for the mean concentrations and correlations were considered statistically significant for *p* values of ≤ 0.05.

### Ethics approval and consent to participate

All procedures performed in studies involving human participants were performed in accordance with the ethical standards of the institutional and/or national research committee and with the 1964 Helsinki declaration and its later amendments or comparable ethical standards. The study was approved by the Institutional Review Board of our hospital (FEMH-IRB-108025-E).

### Consent for publication

The need for patient consent for publication was waived.

## Results

### Effects of different combinations of RT plus sorafenib on PVTT recanalization in HCC patients

The conventional and SBRT groups comprised 25 and 18 patients, respectively. The median age was 62 years (range, 36–85 years), and 84% were men. All had a Child-Pugh score of A with a Barcelona Clinic Liver Cancer (BCLC) classification stage of C. The equivalent doses (EQD_2_) for the conventional and SBRT groups were 52.4 ± 6.8 Gy and 57.1 ± 10.1 Gy (*p* = 0.085), respectively. Under these similar EQD_2_ values, the percentages of complete recanalization in the conventional and SBRT groups were 8% and 28% (*p* = 0.014), respectively (Table 1).

**Table 1:** Hepatocellular carcinoma patients with portal vein tumor thrombosis, Child-Pugh score A and Barcelona Clinic Liver Cancer (BCLC) classification stage C who were treated with sorafenib and conventional radiotherapy (RT, n = 25) or with stereotactic body radiation therapy (SBRT, n = 18). The equivalent dose (EQD_2_) values in the conventional and SBRT groups were 52.4 ± 6.8 Gy and 57.1 ± 10.1 Gy (*p* = 0.085), respectively.

Group	Recanalization (n/total number [%])			P value
	Complete recanalization	Partial recanalization	No response	
Conventional RT (fraction size <5 Gy)	2/25 (8.0%)	13/25 (52.0%)	10/25 (40.0%)	0.014*
SBRT (fraction size ≥ 5 Gy)	5/18 (27.8%)	12/18 (66.7%)	1/18 (5.6%)	

*The p value was verified by Fisher’s exact test.

### Concurrent and sequential sorafenib treatment with irradiation inhibited the viability of Huh-7 cells in a dose-dependent manner

The cytotoxicity of sorafenib in concurrent regimens with RT_2Gy_ and RT_9Gy_ ranged from 2.5 to 20 μM, resulting in increases in cell death from 12.2% to 64.5% and 8.5% to 63.3%, respectively. The cytotoxicity of different concentrations of sorafenib after RT_2Gy_ and RT_9Gy_ resulted in increases in cell death from 15.0% to 70.4% and 13.4% to 78.2%, respectively.

### Effects of irradiation with different time schedules and doses on NF-κB and P-gp activity in Huh-7 cells

**Figure 2:**
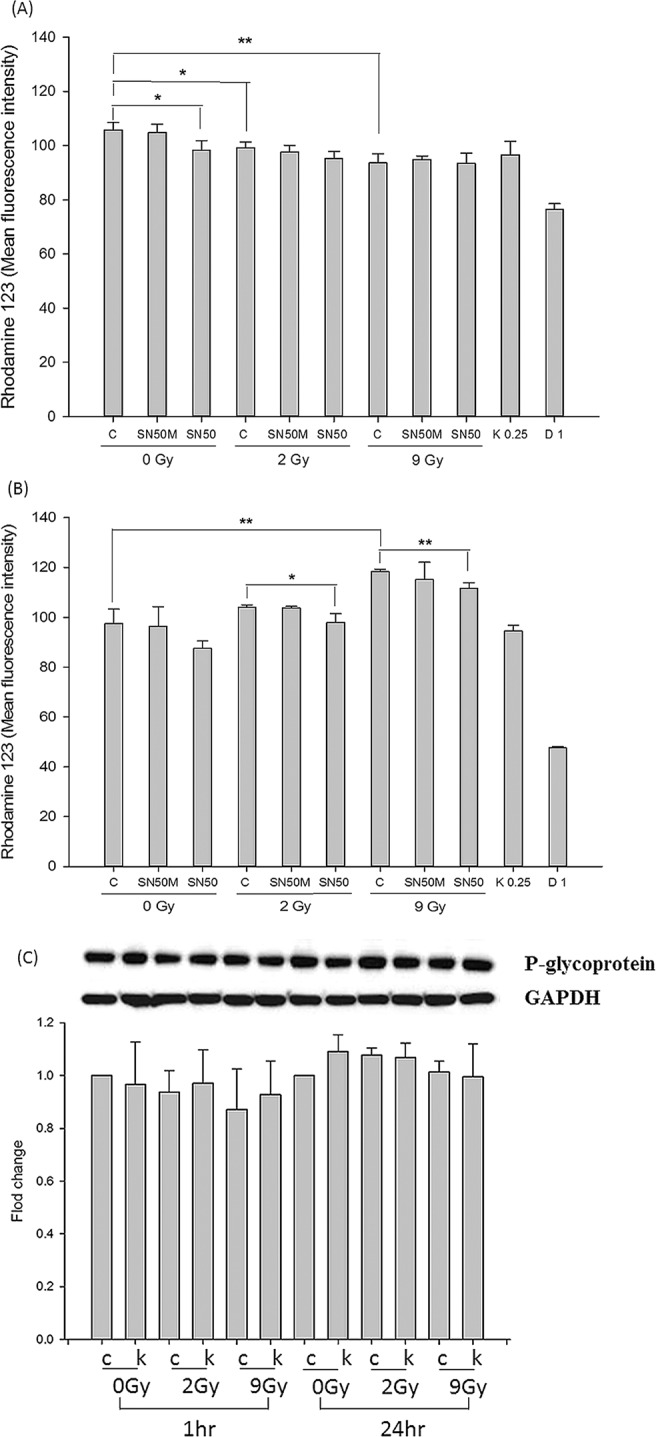
Effects of RT under different time schedules and doses on P-gp activity after pretreatment with 20 μM NF-κB inhibitor (SN50) or inactive peptide control (SN50M) in the Huh 7 cell line. (**A**) RT_2Gy_ and RT_9Gy_ upregulated P-gp activity compared with that in the control group. However, the concurrent RT_2Gy_ and RT_9Gy_ regimens did not alter the Rho-123 intensity in Huh 7 cells through NF-κB. (**B**) The sequential RT_9Gy_ regimen downregulated P-gp activity. Both the sequential RT_2Gy_ and RT_9Gy_ RT regimens decreased P-gp activity through NF-κB. (**C**) Western blot analysis of Huh-7 cells treated with different doses and regimens of RT. GAPDH served as the control in the analyses (C: control; K: 1.25 µM ketoconazole). Data from 3 separate experiments are expressed as the mean ± SEM values (*p* < 0.05: *p < 0.01: **compared with the control group).

Compared to that in the sham group, the Rho-123 intensity in Huh-7 cells was decreased in the group treated with SN50 (an NF-κB inhibitor; 105.8 ± 2.88 vs. 98.3 ± 3.56, *p* = 0.047), indicating that P-gp activity increased after NF-κB activity was inhibited. P-gp activity in Huh 7 cells was correlated with NF-κB activity. Additionally, the concurrent regimen increased P-gp activity by decreasing the Rho-123 intensity (RT_2Gy_: 99.2 ± 2.2, *p* = 0.035 and RT_9Gy_: 93.6 ± 3.4, *p* = 0.009, respectively) compared with that in the control group (Fig. 2A). However, no statistically significant differences were observed for concurrently irradiated Huh-7 cells treated with or without SN50.

In the sequential regimen, the Rho-123 intensity decreased after NF-κB inhibition compared with that after control treatment (87.6 ± 3.0 vs. 97.5 ± 5.8, *p* = 0.056) (Fig. 2B). The Rho-123 intensities in the RT_2Gy_ and RT_9Gy_ groups were 103.8 ± 0.6 (*p* = 0.123) and 118.4 ± 0.9 (*p* = 0.003), respectively. Sequential RT_9Gy_ significantly decreased P-gp activity compared to that in the control group. Interestingly, after pretreatment with SN50, the Rho-123 intensity declined significantly to 98.0 ± 3.5 in the RT_2Gy_ group and 111.7 ± 2.2 in the RT_9Gy_ group compared to that in the corresponding RT_2Gy_ (*p* = 0.046) and RT_9Gy_ (*p* = 0.007) only groups (Fig. 2B). Sequential RT decreased P-gp activity, and this decrease was reversed by treatment with the NF-κB inhibitor. However, Western blot analysis showed no significant differences in P-gp expression in Huh-7 cells treated with or without RT in either regimen (Fig. 2C).

### The concurrent RT regimen increased P-gp activity in freely moving rats

**Figure 3:**
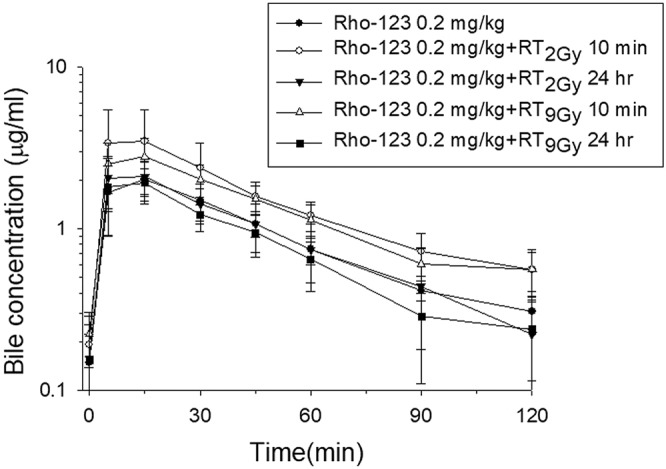
Mean bile concentration-time curve of Rho-123 in rats. Five groups were established: sham RT + Rho-123 (0.2 mg/kg, i.v.) (●); concurrent regimen, RT_2Gy_ + Rho-123 (0.2 mg/kg, i.v.) 10 minutes (min) later (○); sequential regimen, RT_2Gy_ + Rho-123 (0.2 mg/kg, i.v.) 24 hr later (▼); concurrent regimen, RT_9Gy_ + Rho-123 (0.2 mg/kg, i.v.) 10 min later (△); and sequential regimen, RT_9Gy_ + Rho-123 (0.2 mg/kg, i.v.) 24 hr later (■). The data are expressed as the mean ± SEM values (n = 6 per group).

Compared to that in the sham group, the AUC of biliary excretion of Rho-123 increased in both the concurrent RT_2Gy_ and RT_9Gy_ groups, by 69.0% (*p* = 0.013) and 44.2% (*p* = 0.018), respectively. Additionally, the AUC of Rho-123 biliary excretion was decreased for both the sequential RT_2Gy_ and RT_9Gy_ groups compared to the corresponding concurrent groups, by 39.5% (*p* = 0.047) and 38.6% (*p* = 0.017), respectively (Fig. 3, Table 2).

**Table 2:** Estimated biliary excretion pharmacokinetic parameters of Rho-123 (0.2 mg/kg, i.v.) in rats. Five groups were established in both studies: sham RT + Rho-123 (0.2 mg/kg, i.v.); concurrent regimen, RT_2Gy_ + Rho-123 (0.2 mg/kg, i.v.) 10 min later; sequential regimen, RT_2Gy_ + Rho-123 (0.2 mg/kg, i.v.) 24 hr later; concurrent regimen, RT_9Gy_ + Rho-123 (0.2 mg/kg, i.v.) 10 min later; and sequential regimen, RT_9Gy_ + Rho-123 (0.2 mg/kg i.v.) 24 hr later. The data are expressed as the mean ± SEM values (n = 6 per group).

Pharmacokinetic parameter	Rho-123 (0.2 mg/kg)	RT_2Gy_		RT_9Gy_	
		After 10 min + Rho-123 (0.2 mg/kg)	After 24 hr +Rho-123 (0.2 mg/kg)	After 10 min + Rho-123 0.2 mg/kg	After 24 hr + Rho-123 (0.2 mg/kg)
AUC_0-T_ (min*µg/mL)	112.64 ± 23.51	190.38 ± 63.61^a^	115.23 ± 33.31^b,g^	162.42 ± 38.13^c,e^	99.48 ± 27.16^d,f,h^
C_max_ (µg/mL)	2.01 ± 0.60	3.56 ± 1.93	2.24 ± 0.57	2.86 ± 0.83	2.07 ± 0.75
T_max_ (min)	14 ± 4	11 ± 5	9 ± 5	11 ± 5	13 ± 4
t_½_ (min)	42 ± 13	46 ± 13	32 ± 8	43 ± 6	34 ± 9
Cl (mL/min/kg)	1.56 ± 0.30	0.92 ± 0.23	1.73 ± 0.61	1.07 ± 0.33	1.91 ± 0.56
MRT (min)	61 ± 14	66 ± 24	47 ± 12	66 ± 8	50 ± 13

AUC, area under the concentration vs. time curve; Tmax, the time at which Cmax is observed; Cmax, peak plasma concentration of the drug after administration.

^a^RT_2Gy_ (wait 10 min) + Rho-123 (0.2 mg/kg) vs. Rho-123 (0.2 mg/kg) only, *p* = 0.013.

^b^RT_2Gy_ (wait 24 hr) + Rho-123 (0.2 mg/kg) vs. Rho-123 (0.2 mg/kg) only, *p* = 0.877.

^c^RT_9Gy_ (wait 10 min) + Rho-123 (0.2 mg/kg) vs. Rho-123 (0.2 mg/kg) only, *p* = 0.018.

^d^RT_9Gy_ (wait 24 hr) + Rho-123 (0.2 mg/kg) vs. Rho-123 (0.2 mg/kg) only, *p* = 0.391.

^e^RT_2Gy_ (wait 10 min) + Rho-123 (0.2 mg/kg) vs. RT_9Gy_ (wait 10 min) + Rho-123 (0.2 mg/kg), *p* = 0.424.

^f^RT_2Gy_ (wait 24 hr) + Rho-123 (0.2 mg/kg) vs. RT_9Gy_ (wait 24 hr) + Rho-123 (0.2 mg/kg), *p* = 0.436.

^g^RT_2Gy_ (wait 10 min) + Rho-123 (0.2 mg/kg) vs. RT_2Gy_ (wait 24 hr) + Rho-123 (0.2 mg/kg), *p* = 0.047.

^h^RT_9Gy_ (wait 10 min) + Rho-123 (0.2 mg/kg) vs. RT_9Gy_ (wait 24 hr) + Rho-123 (0.2 mg/kg), *p* = 0.017.

### The sequential RT regimen increased CYP3A4 activity in freely moving rats

**Figure 4:**
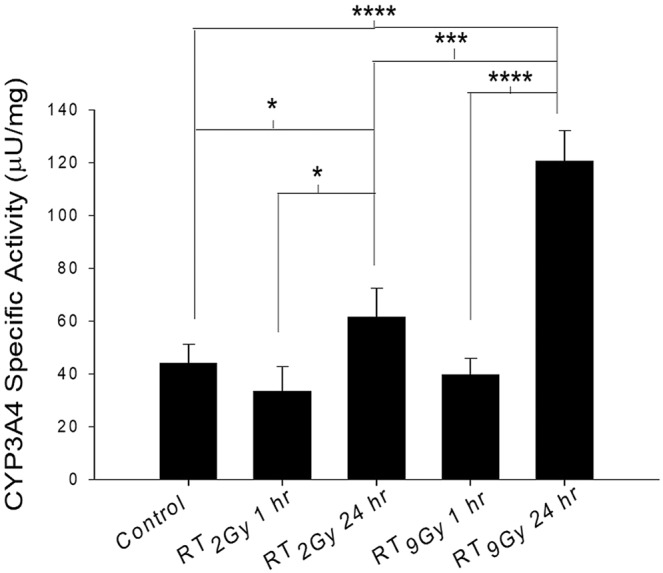
Effects of irradiation on the CYP3A4 protein expression level. CYP3A4 activity was increased significantly by the sequential RT_2Gy_ and RT_9Gy_ regimens compared with the concurrent RT_2Gy_ and RT_9Gy_ regimens of RT. Moreover, compared with RT_2Gy_, RT_9Gy_ significantly increased CYP3A4 activity in the sequential regimen. The data are expressed as the mean ± SEM values (n = 5 per group) (*p* < 0.05: *p < 0.01: **p < 0.005: ***p < 0.001: ****).

Compared to the control regimen, both the sequential RT_2Gy_ and RT_9Gy_ regimens significantly increased CYP3A4 activity by 40.9% (*p* = 0.033) and 175.0% (*p* < 0.001), respectively. In addition, compared with the corresponding concurrent regimens, the sequential RT_2Gy_ and RT_9Gy_ regimens significantly increased CYP3A4 activity by 82.4% (*p* = 0.028) and 202.5% (*p* = 0.0004), respectively. Moreover, compared with RT_2Gy_, RT_9Gy_ significantly increased CYP3A4 activity by 95.2% in the sequential groups (*p* = 0.0029) (Fig. 4).

### Both RT_2Gy_ and RT_9Gy_ modulated the AUC of plasma sorafenib, and these effects were reversed by pretreatment with CsA in freely moving rats

Compared to that in the sham group, the AUC of sorafenib in the concurrent RT_2Gy_ and RT_9Gy_ groups and the sequential RT_9Gy_ group were increased by 131.8% (*p* = 0.046), 162.6% (*p* = 0.038) and 102.4% (*p* = 0.018), respectively. In contrast, the AUC of sorafenib was decreased by 58.8% in the sequential RT_2Gy_ group (*p* = 0.036).

**Figure 5:**
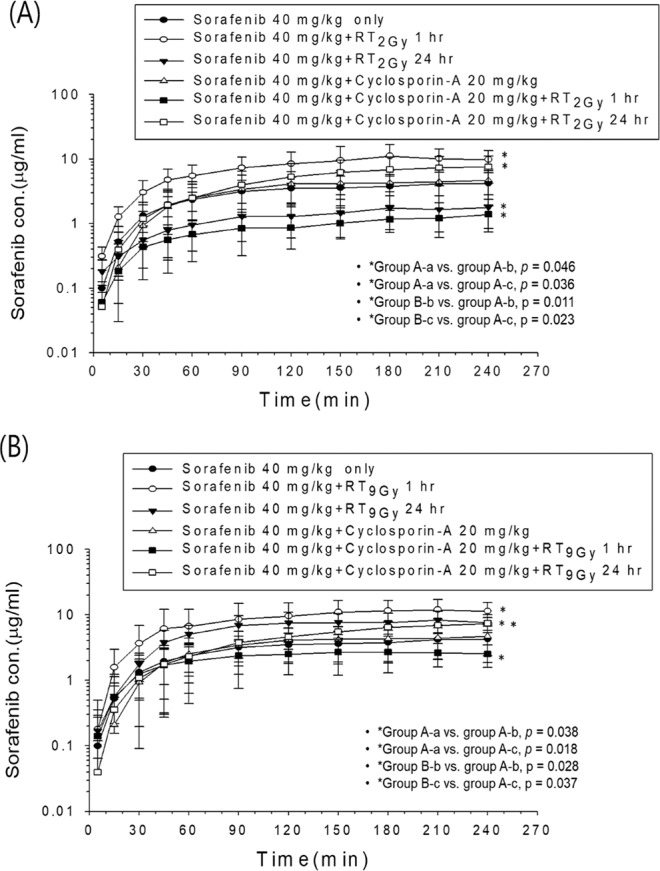
The concentration vs. time curves for sorafenib in the plasma of rats treated under different time schedules with or without irradiation (RT) in (I) the sorafenib only (40 mg/kg, orally (p.o.)) group and (II) the cyclosporine A (CsA, a P-glycoprotein [P-gp] inhibitor, 20 mg/kg, i.p. 30 min before RT) pretreatment group. (**A**) For whole-liver RT at 2 Gy (RT_2Gy_) in group I: (a) sham RT + sorafenib (40 mg/kg) (●); (b) RT_2Gy_ + sorafenib (40 mg/kg, p.o.) 1 hr later (○); (c) RT_2Gy_ + sorafenib (40 mg/kg, p.o.) 24 hr later (▼). For whole-liver RT_2Gy_ in group II: (a) CsA (20 mg/kg, i.p.) + sorafenib (40 mg/kg, p.o.) (∆); (b) CsA (20 mg/kg, i.p.) + RT_2Gy_ + sorafenib (40 mg/kg, p.o.) 1 hr later (■); (c) CsA (20 mg/kg, i.p.) + RT_2Gy_ + sorafenib (40 mg/kg, p.o.) 24 hr later (□). (**B**) For 1.5 × 1.5 cm RT at 9 Gy (RT_9Gy_) in group I: (a) sham RT + sorafenib (40 mg/kg (●); (b) RT_9Gy_ + sorafenib (40 mg/kg, p.o.) 1 hr later (○); (c) RT_9Gy_ + sorafenib (40 mg/kg, p.o.) 24 hr later (▼). For 1.5 × 1.5 cm RT_9Gy_ in group II: (a) CsA (20 mg/kg, i.p.) + sorafenib (40 mg/kg, p.o.) (∆); (b) CsA (20 mg/kg, i.p.) + RT_9Gy_ + sorafenib (40 mg/kg, p.o.) 1 hr later (■); (c) CsA (20 mg/kg, i.p.) + RT_9Gy_ + sorafenib (40 mg/kg, p.o.) 24 hr later (□). The data are expressed as the mean ± SEM values (n = 6 per group).

For the concurrent regimen, the AUC in the CsA pretreatment plus sorafenib group was decreased by 83.3% compared to that in the RT_2Gy_ plus sorafenib group (*p* = 0.011). For the sequential regimen, pretreatment with CsA increased the AUC by 274.7% (*p* = 0.023). The AUC in the pretreatment with CsA plus RT_9Gy_ concurrent with sorafenib group was decreased by 68.1% compared with that in the RT_9Gy_ concurrent with sorafenib group (*p* = 0.028). Similarly, the AUC was decreased by 47.0% in the pretreatment with CsA plus RT_9Gy_ followed by sorafenib group compared with that in the sequential RT_9Gy_ group (*p* = 0.037). The data are presented in Fig. 5, Tables 3 and 4.

**Table 3:** Estimated pharmacokinetic parameters of sorafenib with or without RT_2Gy_ in rats after (I) treatment with sorafenib alone (40 mg/kg, p.o.) and (II) pretreatment with cyclosporin A (CsA, a P-glycoprotein [P-gp] inhibitor, 20 mg/kg, i.p., 30 min before RT).

PK parameter	Sorafenib (40 mg/kg) only	RT_2Gy_		CsA (20 mg/kg, i.p.) + sorafenib (40 mg/kg)	CsA (20 mg/kg) i.p.+ RT_2Gy_	
		After 1 hr + sorafenib (40 mg/kg)	After 24 hr+ sorafenib (40 mg/kg)		After 1 hr + sorafenib (40 mg/kg)	After 24 hr + sorafenib (40 mg/kg)
AUC_0-T_ (min*µg/mL)	719.5 ± 348.6	1669.0 ± 958.7^a^	296.9 ± 169.8^b^	784.5 ± 456.7	277.7 ± 182.9^c,e^	1113.3 ± 608.0^d,f^
T_max_ (min)	225 ± 16	175 ± 40	204 ± 25	240 ± 0	198 ± 66	225 ± 30
C_max_ (µg/mL)	4.39 ± 2.28	10.14 ± 5.93	1.89 ± 1.08	4.69 ± 2.31	1.72 ± 0.96	7.71 ± 3.41

The data are expressed as the mean ± SEM values (n = 6). AUC, area under the concentration vs. time curve; Tmax, the time at which Cmax is observed; Cmax, peak plasma concentration of the drug after administration.

^a^RT_2Gy_ + sorafenib (40 mg/kg, p.o.) 1 hr later vs. sham RT + sorafenib (40 mg/kg), *p* = 0.046.

^b^RT_2Gy_ + sorafenib (40 mg/kg, p.o.) 24 hr later vs. sham RT + sorafenib (40 mg/kg), *p* = 0.036.

^c^CsA (20 mg/kg, i.p.) + RT_2Gy_ + sorafenib (40 mg/kg, p.o.) 1 hr later vs. sham RT + sorafenib (40 mg/kg), *p* = 0.032.

^d^CsA (20 mg/kg, i.p.) + RT_2Gy_ + sorafenib (40 mg/kg, p.o.) 24 hr later vs. sham RT + sorafenib (40 mg/kg), *p* = 0.224.

^e^CsA (20 mg/kg, i.p.) + RT_2Gy_ + sorafenib (40 mg/kg, p.o.) 1 hr later vs. RT_2Gy_ + sorafenib (40 mg/kg, p.o.) 1 hr later, *p* = 0.011.

^f^CsA (20 mg/kg, i.p.) + RT_2Gy_ + sorafenib (40 mg/kg, p.o.) 24 hr later vs. RT_2Gy_ + sorafenib (40 mg/kg, p.o.) 24 hr later, *p* = 0.023.

**Table 4:** Estimated pharmacokinetic parameters of sorafenib with or without RT_9Gy_ in rats after (I) treatment with sorafenib alone (40 mg/kg, p.o.) and (II) pretreatment with cyclosporin A (CsA, a P-glycoprotein [P-gp] inhibitor, 20 mg/kg, i.p., 30 min before RT).

PK parameters	Sorafenib (40 mg/kg) only	RT_9Gy_		CsA (20 mg/kg, i.p.) + sorafenib (40 mg/kg)	CsA (20 mg/kg) i.p.+ RT_9Gy_	
		After 1 hr + sorafenib (40 mg/kg)	After 24 hr + sorafenib (40 mg/kg)		After 1 hr + sorafenib (40 mg/kg)	After 24 hr + Sorafenib (40 mg/kg)
AUC_0-T_ (min*µg/mL)	719.5 ± 348.6	1891.0 ± 1151.6^a^	1456.5 ± 534.4^b^	784.5 ± 456.7	604.0 ± 421.3^c,e^	772.4 ± 449.5^d,f^
T_max_ (min)	225 ± 16	195 ± 35	185 ± 40	240 ± 0	200 ± 56	235 ± 12
C_max_ (µg/mL)	4.39 ± 2.28	12.26 ± 6.30	8.686 ± 2.73	4.69 ± 2.31	3.74 ± 2.17	5.43 ± 3.22

The data are expressed as the mean ± SEM values (n = 6). AUC, area under the concentration vs. time curve; Tmax, the time at which Cmax is observed; Cmax, peak plasma concentration of the drug after administration.

^a^RT_9Gy_ + sorafenib (40 mg/kg, p.o.) 1 hr later vs. sham RT + sorafenib (40 mg/kg), *p* = 0.038.

^b^RT_9Gy_ + sorafenib (40 mg/kg, p.o.) 24 hr later vs. sham RT + sorafenib (40 mg/kg), *p* = 0.018.

^c^CsA (20 mg/kg, i.p.) + RT_9Gy_ + sorafenib (40 mg/kg, p.o.) 1 hr later vs. sham RT + sorafenib (40 mg/kg), *p* = 0.616.

^d^CsA (20 mg/kg, i.p.) + RT_9Gy_ + sorafenib (40 mg/kg, p.o.) 24 hr later vs. sham RT + sorafenib (40 mg/kg), *p* = 0.825.

^e^CsA (20 mg/kg, i.p.) + RT_9Gy_ + sorafenib (40 mg/kg, p.o.) 1 hr later vs. RT_9Gy_ + sorafenib (40 mg/kg, p.o.) 1 hr later, *p* = 0.028.

^f^CsA (20 mg/kg, i.p.) + RT_9Gy_ + sorafenib (40 mg/kg, p.o.) 24 hr later vs. RT_9Gy_ + sorafenib (40 mg/kg, p.o.) 24 hr later, *p* = 0.037.

### Distribution of sorafenib in the organs of rats treated with different regimens of RT and sorafenib

**Figure 6:**
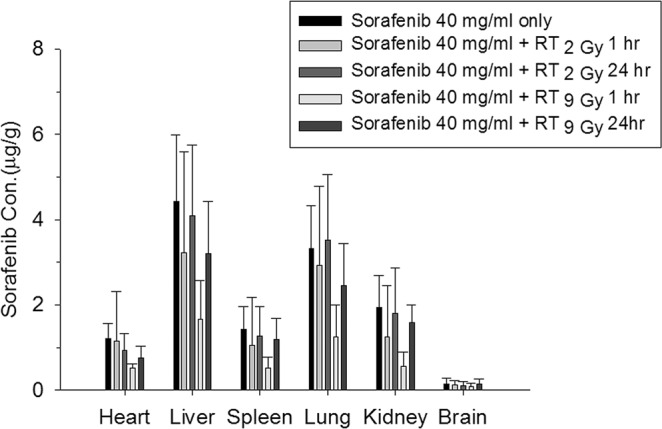
The concentrations (µg/g) of sorafenib in different organs were measured 4 hr after oral administration. The regimens were RT_2Gy_ or RT_9Gy_ concurrent or sequential with or without 40 mg/kg sorafenib. (n = 5 per group; *p* < 0.05: *, *p* < 0.01: **).

As Fig. 6 shows, the sorafenib concentration in various organs of rats in the sorafenib only (sham) group decreased in the following order: liver (4.43 ± 1.56 μg/g) > lungs (3.20 ± 1.00 μg/g) > kidneys (1.93 ± 0.75 μg/g) > spleen (1.42 ± 0.53 μg/g) > heart (1.20 ± 0.36 μg/g) > brain (0.14 ± 0.15 μg/g). The distributions of sorafenib in rats treated with RT_2Gy_ and RT_9Gy_ under the sequential regimen did not differ significantly from those in sham group rats. However, RT_9Gy_ concurrent with sorafenib significantly decreased the level of sorafenib in all organs except the brain (Fig. 6). Thus, the sequential regimen was associated with a greater biodistribution than the concurrent regimen.

## Discussion

Several studies have reported a reasonable combination of sorafenib with RT or SBRT^11,24^. The Radiation Therapy Oncology Group (RTOG) launched a randomized phase III trial (RTOG 1112) to study the effect of sorafenib alone vs. SBRT followed by sorafenib in HCC. In a case report, the HCC tumor volume was reduced by 39% under the combination regimen^24^. Additionally, a complete or partial response rate of 55% was observed for HCC patients treated with a combination of sorafenib and RT^11^. However, 30–66% of patients with HCC treated with SBRT concurrently or sequentially with sorafenib were reported to experience grade 3 or greater dose-limiting toxicities^12,13,25^. These studies suggest interactions between RT and sorafenib. Here, under similar EQD_2_ values, PVTT recanalization was 3-fold more efficient with SBRT combined with sorafenib than with conventional RT (*p* = 0.014). However, the interactions between sorafenib and different RT techniques are still unclear and may limit the application of such combinations.

Studies have suggested varied time schedules for RT and sorafenib regimens. In HCT116 xenograft tumor growth delay experiments^26^ and HCC cell line studies^27,28^, RT followed by sorafenib resulted in the greatest delay in tumor growth and efficacy against HCC cell lines, respectively. However, tumor growth delays in xenograft experiments with concurrent regimens have also been reported^27^. The results of both a phase I study^12^ and clinical activity in patients support the superiority of the sequential regimen^24,29^. In the current study, concurrent RT_2Gy_ and RT_9Gy_ regimens and a sequential of RT_9Gy_ regimen increased the AUC of sorafenib. In contrast, sequential RT_2Gy_ decreased the AUC of sorafenib. Both the concurrent and sequential RT_9Gy_ regimens increased the concentration of sorafenib by more than 2-fold compared with that observed for sorafenib alone. Furthermore, dose-dependent sorafenib cytotoxicity was noted in Huh-7 cells. In other words, a heretofore unreported RT-PK phenomenon between RT and sorafenib was observed. Additionally, high-dose irradiation more efficiently modulated the PK of sorafenib than conventional-dose irradiation in both the sequential and concurrent regimens.

The elimination half-life of sorafenib is approximately 25–48 hr; sorafenib accounts for approximately 70–85% of the steady-state circulating analytes in plasma^30^ and binds with moderate affinity to the efflux transporter P-gp^31^. The permeability of sorafenib across the gastrointestinal (GI) epithelium was found to be high, and sorafenib was found to enter the enterohepatic circulation^32^. Approximately 50% of an orally administered dose of sorafenib is recovered as unchanged drug by biliary excretion or lack of absorption^33^. In fact, the enterohepatic circulation also increases the utility of sorafenib in the body^34^. the findings of the present study confirm that either low- or high-dose irradiation, in a concurrent regimen acts as a P-gp inducer, possibly accelerating the excretion of sorafenib from the liver into the GI tract. In other words, sorafenib treatment concurrent with RT may cause sorafenib to reenter the systemic circulation, thus increasing its AUC.

An attractive strategy to improve drug delivery and overcome drug resistance is inhibition of P-gp. The expression level of P-gp is variably reported to show no change^35^, a decrease^36^, or an increase^37^ responses to irradiation. Variable activity of P-gp after the different regimens of RT was noted in the current study. Rho-123 efflux is P-gp–dependent and has been used extensively to assess efflux from drug-resistant cell lines expressing P-gp^38^. Concurrent RT decreased the Rho-123 intensity, suggesting that concurrent RT upregulates P-gp activation. Radiation-induced apoptosis is inhibited by the expression of P-gp; however, the effect on radioresistance may be limited by simultaneously increasing mitotic catastrophe and senescence in radiation-damaged cells^36^.

In contrast, the Rho-123 intensity was increased by the sequential regimen, suggesting that sequential RT downregulates P-gp activity. However, the expression of P-gp in cells treated with the different regimens either with or without RT did not differ. In other words, the RT regimen affects the activity but not the expression of P-gp. Sorafenib is a moderate-affinity P-gp substrate^39^. A study in sorafenib-resistant HCC cell lines confirmed that sorafenib resistance could be mediated through upregulation of P-gp^40^. In addition, RT followed by sorafenib was noted to produce the greatest delay in HCT116 xenograft tumor growth^26^. In the current study, the concurrent RT_2Gy_ and RT_9Gy_ regimens and sequential RT_9Gy_ regimen increased the AUC of sorafenib. However, the sequential RT_2Gy_ regimen decreased the concentration of sorafenib. These findings suggest that the sequential regimen of SBRT plus sorafenib might be more suitable than the concurrent regimen.

NF-κB is a radiation-responsive transcription factor^41^, and P-gp activation has been demonstrated to occur through NF-κB activation^42^. In the current study, the sequential RT regimen downregulated P-gp activity, and pretreatment with the NF-κB inhibitor followed by the sequential regimen exhibited the opposite effect on P-gp activity. Sorafenib potently inhibits P-gp-mediated multidrug resistance (MDR) by inhibiting MAPK/ERK pathway signaling in HCC^43^. Additionally, blocking the phosphoinositide 3-kinase (PI3K)/Protein kinase B (PKB, or Akt) signaling pathway enhances the efficacy of sorafenib^44^. NF-κB inhibits ERK activation to enhance cell survival^45^. In addition, crosstalk occurs between the PI3K/AKT and MAPK/ERK pathways^46^. Therefore, the results of the current study suggest that RT and RT regimens impact P-gp activation and may act through NF-κB via complex pathways. In addition, the sequential RT_9Gy_ regimen may have benefits for HCC patients; however, more data is required for confirmation.

Sorafenib is metabolized to sorafenib N-oxide, undergoing oxidative metabolism via CYP3A4^47^. Notably, the AUC of sorafenib was reduced by an average of 37% with concomitant administration of the CYP3A4 inducer rifampicin^47^. Additionally, NF-𝜅B activity may provide a feedback mechanism that regulates ROS production by CYP3A4, and inhibition of this activity reduces the protein stability of CYP3A4^48^. Additionally, compared to the concurrent regimen, both the sequential RT_2Gy_ and RT_9Gy_ regimens significantly increased CYP3A4 activity and decreased the AUC of sorafenib. However, the sequential RT_9 Gy_ regimen increased the AUC of sorafenib approximately twofold compared to that observed for sham RT. Therefore, the sequential RT regimen increased CYP3A4 activity to modulate the PK of sorafenib. With respect to RT-sorafenib synergy on tumor response, overall sorafenib activity and also with respect to potential for increased risk of normal tissue toxicity, the sequential RT_9Gy_ regimens are more suitable than the concurrent regimen for patients with HCC.

Plastaras *et al*.^26^ reported that RT followed by sorafenib was associated with the greatest tumor growth delay; however, they used an HCT116 (colon cancer) xenograft model. Brade *et al*.^12^ suggested that concurrent SBRT with sorafenib may cause unpredictable toxicity and that such a regimen should be used with caution. The current data suggest that the liver, lung and kidneys are the primary organs in which sorafenib accumulates. Additionally, the sequential regimen was associated with a greater biodistribution than the concurrent regimen, providing a rationale for the treatment of patients with organ metastasis with the sequential regimen of RT and sorafenib. The lowest level of sorafenib was detected in the brain regardless of administration of RT and regardless of the dose or regimen, indicating that sorafenib has difficulty permeating the blood-brain barrier and that local RT to the liver cannot modulate the level of sorafenib in the brain. Compared to the sham RT, Sequential RT_9Gy_, as well as concurrent RT_2Gy_ and RT_9Gy_, increased the AUC of sorafenib, further supporting SBRT followed by sorafenib as the optimized regimen.

**Figure 7:**
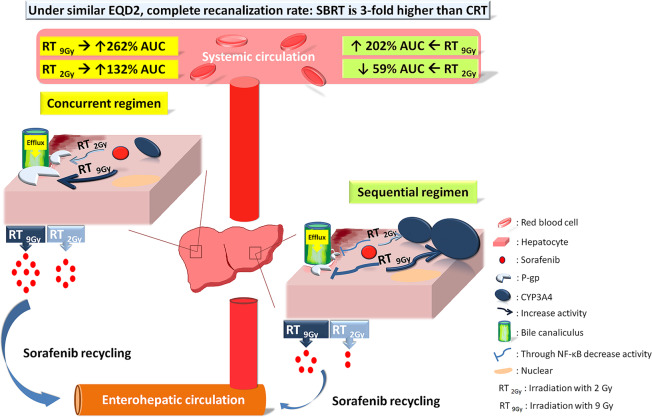
Schematic representation of the RT-PK phenomenon of sorafenib. When the liver is irradiated concurrently with sorafenib administration, the activity of P-gp is upregulated to increase efflux activity and to intensify the recycling and enterohepatic circulation of sorafenib, which in turn increases the AUC of sorafenib. In contrast, the sequential RT regimen increases the expression of CYP3A4, which increases the oxidative metabolism of sorafenib. Additionally, compared with sorafenib alone, RT_9Gy_ increased the AUC twofold; thus, SBRT is more efficient than the conventional RT technique to increase the rate of recanalization in HCC patients with PVTT under treatment with RT and sorafenib.

These lines of evidence support the dynamic processes in the RT-PK phenomenon of sorafenib. When the liver is irradiated with concurrent administration of sorafenib, the activity of P-gp is upregulated to increase efflux activity and to intensify the recycling and enterohepatic circulation of sorafenib, which in turn increases the AUC of sorafenib. In contrast, under sequential irradiation regimens, P-gp activity is downregulated through NF-κB, possibly decreasing sorafenib efflux. In addition, the expression of CYP3A4 is increased under sequential regimens, which increases the oxidative metabolism of sorafenib. Additionally, compared with sorafenib alone, RT_9Gy_ increased the AUC of sorafenib twofold, and RT_2Gy_ increased it 1.3-fold; thus, RT_9Gy_ was more efficient than RT_2Gy_. Clinically, compared with conventional techniques, SBRT increased the rate of recanalization with similar EQD_2_ values for HCC patients with PVTT treated with sorafenib. Therefore, the optimized regimen for HCC patients might be SBRT followed by sorafenib (Fig. 7).

The current study had some limitations. First, the pharmacodynamics of sorafenib during RT were not modeled, although the AUC of sorafenib was increased by concurrent RT_2Gy_ and RT_9Gy_ and by sequential RT_9Gy_. However, the clinical reports support the effects of the RT-PK phenomenon of sorafenib combinations^11,12,24,29^. Second, we retrospectively reviewed the clinical data without dividing the patients into the concurrent and sequential groups by clinical experience and supporting the superiority of the SBRT combination. Here, the obviously increased AUC of sorafenib was consistent between the RT_9Gy_ regimen and the conventional dose, indirectly supporting the clinical observations. Third, RT was delivered with sorafenib in the current study in a single fraction. However, the clinical practice is to deliver RT in continuous daily fractions or multiple fractions either concurrent or sequential with sorafenib. Further study to recapitulate clinical practice by using multiple fractions with different RT regimens is warranted in order to exploit the RT-PK phenomenon to optimize the timing, duration, and dosing of sorafenib for the design of clinical trials.

## Conclusions

To our knowledge, our study is the first to show that the PK of sorafenib can be dynamically modulated by irradiation, supporting the RT-PK phenomenon between RT and sorafenib. Concurrent RT increased P-gp activity, and sequential RT decreased P-gp activity. However, the expression of P-gp was not affected by different RT regimens. Additionally, sequential RT increased CYP3A4 activity. The AUC of sorafenib was increased twofold by high-dose irradiation, providing new insight into why high-dose irradiation is a more effective approach than conventional RT for modulating the PK of sorafenib and indicating especially that the cytotoxicity of sorafenib is correlated with the dose. Additionally, SBRT followed by sorafenib could be the optimized regimen. These data provide insight into the design of clinical trials combining RT and targeted therapeutics.

## Data Availability

The datasets used and/or analyzed are available from the corresponding author upon reasonable request.
